# Microwave-enhanced antibacterial activity of polydopamine–silver hybrid nanoparticles†

**DOI:** 10.1039/d3ra07543e

**Published:** 2024-03-11

**Authors:** Swetha Lingamgunta, Yao Xiao, Heungjae Choi, Graham Christie, Ljiljana Fruk

**Affiliations:** a Department of Chemical Engineering and Biotechnology, University of Cambridge Cambridge UK lf389@cam.ac.uk; b School of Engineering, Cardiff University Wales UK

## Abstract

The ever-increasing risks posed by antibiotic-resistant bacteria have stimulated considerable interest in the development of novel antimicrobial strategies, including the use of nanomaterials that can be activated on demand and result in irreversible damage to pathogens. Microwave electric field-assisted bactericidal effects on representative Gram-negative and Gram-positive bacterial strains were achieved in the presence of hybrid polydopamine–silver nanoparticles (PDA–Ag NPs) under low-power microwave irradiation using a resonant cavity (1.3 W, 2.45 GHz). A 3-log reduction in the viability of bacterial populations was observed within 30 minutes which was attributed to the attachment of PDA–Ag NPs and associated membrane disruption in conjunction with the production of intra-bacterial reactive oxygen species (ROS). A synergistic effect between PDA and Ag has been demonstrated whereby PDA acts both as an Ag NP carrier and a microwave enhancer. These properties together with the remarkable adhesivity of PDA are opening a route to design of antibacterial adhesives and surface coatings for prevention of biofilm formation.

## Abstract 2

Ag containing hybrid nanomaterials can be used to induce microwave-guided deactivation of bacterial pathogens.

## Graphical abstract

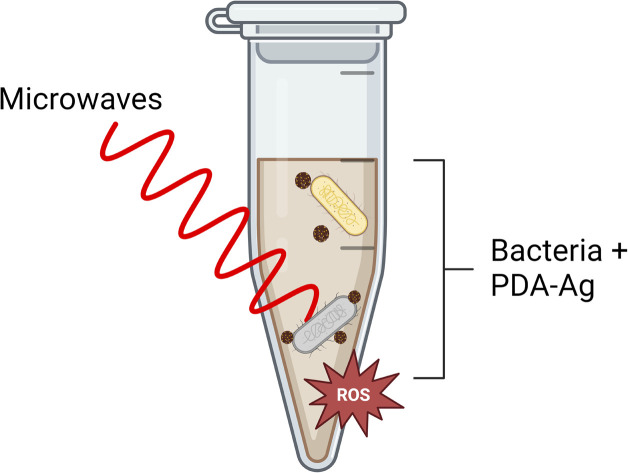

## 1. Introduction

Antibiotic-resistant bacterial strains pose a significant threat to public health and in-patient care in hospitals, particularly for those patients undergoing surgical procedures heavily relying on antibiotic efficacy for recovery. Considering the forecasted 10 million yearly deaths by 2050 linked to bacterial resistance,^1^ it is not surprising that significant efforts have been made into finding alternative approaches to eliminate bacteria.

One of the explored approaches is the use of nanostructured materials, which can act as antibacterial agents either by inducing damage through direct interactions with pathogens^2,3^ or indirectly through production of damaging reactive oxygen species (ROS) or localised thermal effects. The latter can result in the disruption of electrolyte balance, protein denaturation and deregulation of enzymatic activity and gene expression.^4,5^ Nanostructured materials, such as nanoparticles (NPs) have unique properties such as controllable size, tuneable shape and a range of physiochemical characteristics that make them suitable for design of antibacterial agents. Generally, antibacterial nanoparticles can be grouped into three families:^6^ (1) those such as Ag^+^-releasing nanoparticles that act directly on cell integrity, protein activity or gene expression due to their intrinsic properties,^7^ (2) those that act as nanocarriers of other antibacterial agents^8,9^ and (3) those that can be activated by external stimuli such as light. Both organic such as peptide, protein or polymeric NPs,^10^ and inorganic NPs ranging from Ag NP to more complex nanocomposites have successfully been used as antibacterial agents.^5,7,11^ Furthermore, novel nanostructures are continuously reported. For example, nanoparticle-pinched polymer brushes consisting of silica nanospheres (50 nm) grafted with hydrophilic polymers were developed that demonstrated selective toxicity towards bacteria but not mammalian cells due to the ability to remodel bacterial membrane.^12^

However, the most interesting developments in the realm of antibacterial nanostructures stem from stimuli-responsive NPs offering the on-demand activation and usually, temporal and spatial control over antimicrobial activity.^13^ Various hybrids of semiconducting nanoparticles such as TiO_2_ (ref. 14) or ZnO^15^ are widely used for antibacterial applications, although recently, carbon-based structures that can be activated with a range of wavelengths were reported.^16,17^ Although inorganic materials have traditionally been more extensively explored as stimuli-activated systems, organic composites based on conductive polymers are gaining traction as they offer more flexibility in terms of biodegradation, manufacturing, and tunability of irradiation wavelength.^18^ However, the major obstacle in the use of light for NP activation is the limited depth of light penetration,^19^ which confines their application to thin surface coatings or surface-exposed areas of wounds.

Microwaves (MW) have successfully been used in tumour therapy,^20^ and are considered a powerful stimuli for activation of antimicrobial agents.^21^ MWs are components of the electromagnetic spectrum, a form of non-ionising radiation with frequencies ranging from 300 MHz to 300 GHz and wavelengths between 1 m and 1 mm. Due to their longer wavelengths, they offer greater tissue penetration depth. For example, commonly used 2.45 GHz microwaves reach up to 2–3 cm. In addition, lower frequency MWs cause negligible damage to healthy cells and tissues, which makes them suitable for biomedical applications.

Generally, the effects of MWs on bacteria can be classified into thermal or non-thermal, and their activity is both energy and time dependent.^22^ MW-induced thermal effects have already found application in sterilisation as they lead to faster heating and heat-induced bacterial inactivation. On the other hand, the non-thermal MW effects have been shown to lead to destruction of microorganisms at temperatures below the thermal destruction point, which is believed to result from cell membrane disruption and leakage of intracellular material.^21,23,24^ Prolonged exposure to microwave radiation results in increased membrane permeability through formation of pores.^25^ Such pore formation depends on the microwave properties (frequency, pulse width and length, amplitude, and duration of exposure)^26^ and damages microorganisms by compromising their membrane integrity and facilitating the uptake of various antimicrobial molecules or nanoparticles.^27^

Considering the enhanced uptake of nanoparticles under MW irradiation, as well as the use of different NP classes as MW enhancers, we hypothesised that the synergistic effects of rationally designed nanoparticles and low frequency MWs should lead to improved antibacterial activity. Although NPs such as iron oxide (Fe_3_O_4_) NP, metal-doped multiwalled carbon nanotubes, gold (Au) and zirconium oxide (ZrO_2_) were used in MW-enhanced hyperthermia treatment of tumours,^28^ we decided to focus on Ag NPs. These NPs have already been shown to possess broad spectrum antibacterial properties^3^ and could significantly improve MW absorption when doped into non-absorbing materials.^28,29^ Such enhancement has been attributed to the increase in dipole polarization, interface polarization and conductivity loss, all of which ultimately result in an improved energy transfer.^30,31^

Herewith, we describe the preparation of hybrid NPs composed of Ag NPs embedded within a polydopamine (PDA) nanocarrier, a eumelanin-like biopolymer, which has previously been shown to act as MW enhancer with an effective absorption bandwidth of 2.45 GHz, which makes it suitable for use of low frequency MW.^32–34^ In addition to this favourable property, PDA is a suitable carrier for Ag NPs, effectively preventing their undesired aggregation, and it is known for its exceptional adhesive properties.^35,36^ This quality could facilitate interaction with the bacterial cell envelope, thereby encouraging membrane disruption and consequently, the inactivation of bacterial. Our hybrid PDA–Ag NPs show improved MW-guided antibacterial activity, which we attribute to the combination of membrane disruption and intra-bacterial ROS production.

## 2. Experimental

### 2.1 General

All materials were purchased from either Acros Organics, Alfa Aeser, Sigma-Aldrich, or TCI Chemicals in the highest purity available and used without further purification. Ag NPs used for control experiments were obtained from Sigma (20 nm, in a sodium citrate stabiliser). UV-Vis absorption spectra were obtained with an Agilent Cary 300 Spectrophotometer. DLS and zeta-potential measurements were recorded using a Zetasizer Nano Range instrument (Malvern Panalytical). Samples were suspended in water and drop cast on lacey carbon copper grids (Agar Scientific). Thermo Scientific (FEI) Talos F200X G2 TEM was used to obtain images and determine the size of the PDA and Ag nanoparticles. For particle size analysis, sample size is *n* = 25. For bacterial images, grids were glow discharged prior to adding the sample and stained with uranyl acetate. Induced coupled plasma mass spectroscopy (ICP-MS) was used to determine the silver content in the final sample of PDA–silver-NP using the Thermo Fisher Scientific iCAP 7400 ICP-OES Analyser.

### 2.2 Synthesis of polydopamine (PDA)

Aqueous ammonia solution (2.0 mL, 28%) and Milli Q water (90 mL) were mixed with ethanol (40 mL). Dopamine hydrochloride (0.5 g) was dissolved in deionised water (10 mL) and injected into the mixture. The reaction was left to proceed for 12 hours with mild stirring. Large aggregates were removed by centrifugation at 4000 rpm for 10 minutes. PDA-NPs were obtained by centrifugation at 18 rpm for 30 minutes and washed four times. Estimated yield of the PDA-NPs is 50 mg; exact concentration was determined by freeze drying using mannitol as a cryoprotectant. Dried PDA was then washed by centrifugation with water three times and resuspended in water for a final concentration of 1 mg mL^−1^.

### 2.3 Formation of hybrid polydopamine–silver nanoparticles (PDA–Ag)

PDA-NPs (1 mL, 1 mg mL^−1^) were mixed with AgNO_3_ (1 mL, 1 g mL^−1^) and deionised water (8 mL). The reaction was left to proceed for 1 h with mild stirring at room temperature. The resulting solution was washed three times by centrifugation with water. Concentration was determined by freeze-drying 15 mL of PDA–Ag.

### 2.4 Exploration of antibacterial activity

The *E. coli* (top 10) has been transformed to be resistant to β-lactam antibiotics, including carbenicillin. *B. subtilis* (ATCC 6633) has an active β-lactamase, and thus is resistant to β-lactam antibiotics. β-Lactam antibiotics are a group of antibiotics that include penicillins and cephalosporins, which are covalent inhibitors that target bacterial penicillin-binding proteins and disrupt peptidoglycan synthesis.^37^

Upon addition of PDA–Ag or PDA to bacterial suspensions, the suspension turned to a dark brown. Thus, it was not possible accurately monitor the turbidity of the suspensions. Therefore, in this report, the antibacterial efficacy of PDA–Ag is evaluated by the inhibition rate experiment on LB agar plates, and through minimum bactericidal concentration rather than minimum inhibitory concentration (MIC).

### 2.5 Minimum bactericidal concentration (MBC)

The bacterial inoculums were adjusted to 10^8^ CFU mL^−1^ (0.5 McFarland standard). The MBC was defined as the lowest concentration of the antibacterial agents that completely kill the bacteria. 100 μL of PDA–Ag was added to 100 μL of bacterial inoculum in Mueller–Hinton Broth (MHB). After incubation at 37 °C for 24 h, the solutions were washed times with 2× in PBS at 2500 RPM for 7 minutes to remove desorbed antibacterial agents. The pellets were re-suspended in PBS. 100 μL of each solution was spread onto MHA plates and incubated at 37 °C for 24 h. The lowest concentration with no visible growths on the MHA plate was taken as MBC value.

### 2.6 Zone of inhibition

The antibacterial activity of PDA–Ag in the presence of *E. coli* K12 (top 10, carbenicillin resistant) was carried out using the Stokes disc diffusion method. 100 μL of overnight bacterial suspension (adjusted to 0.5 McFarland standard, confirmed using OD600) spread onto Mueller–Hinton agar (MHA). Sterile blank antimicrobial susceptibility discs were loaded with 10 μL PDA–Ag (0.15 mg mL^−1^), 10 μL PDA (0.15 mg mL^−1^) and or 10 μL Ag NP (0.01 mg mL^−1^, matched by ICP analysis). The solutions were dropped onto discs using a pipette, then placed on inoculated agar plates. The plates were then incubated at 37 °C and the zone of inhibition was observed after 24 h of incubation.

### 2.7 Bacterial viability studies

Bacterial viability was performed using Luria–Bertani (LB) agar. For an overnight bacterial culture, 10 μL of bacterial stock was added to 10 mL LB broth and incubated at 37 °C on a 227 RPM orbital shaker. 200 μL solutions of 0.1 PDA–Ag were incubated with OD 1.0 of (1 × 10^8^) laboratory standard *E. coli* (top 10, carbenicillin resistant) and *B. subtilis* (ATCC 6633) in ambient conditions (standard laboratory lighting and temperature) or exposed to microwaves for 0, 2, 5, 30 or 60 minutes. 200 μL solutions were placed in an Eppendorf tube in a bespoke portable microwave applicator (PMA).

The PMA consists of a microwave signal generator, a power amplifier, a resonant cavity, and signal monitoring circuits that measures power delivered to the cavity and reflected back. The Eppendorf tube filled with sample is placed at the centre of the resonant cavity where the electric field is at its maximum. Then the input coupling loop of the resonant cavity was adjusted so that the reflected microwave signal became minimized, which is called a critical coupling condition. 1.3 W of MW power was applied to a critically-coupled resonant cavity, therefore it can be assumed that most of the applied power was delivered to the sample in the form of electric field intensity. To avoid microwave thermal effects, the applied MW signal was chosen to be pulsed at 15% duty ratio over a period of 1 second. In other words, the MW was ON for 150 ms, then OFF for 850 ms over 1 second period with the total exposure time varied over 0, 2, 5, 30 or 60 minutes. The temperature was maintained below 37 °C during MW exposure as shown in ESI Fig. S3.† Cells without microwave treatment were considered as controls. The plates were incubated for 18 hours at 37 °C to measure colony forming units (CFU).

### 2.8 Temperature measurements

The Pico Technology TC-08 thermocouple data logger was used in conjunction with PicoLog for acquisition of temperature changes during microwave experiments. Measurements were taken every 1 second for the entire 1 hour experimental period. Extra care was taken when the thermocouple was inserted into the Eppendorf tube to avoid any interference between the electrical wires of the thermocouple and the electromagnetic field distribution within the resonant cavity.

### 2.9 Bacterial ROS production

Intracellular ROS production was monitored using cell permeable ROS indicator 2′,7′–dichlorofluorescin diacetate which measures hydroxyl, peroxyl, hydrogen peroxide and other reactive oxygen species. Intracellular esterases cleave the acetate groups, trapping indicator within the cell, which can be oxidised by ROS to form the fluorescent DCF. 100 μL of sample was added to a 96-well plate, mixed with 10 μM DCFH-DA and incubated for 5 minutes. The samples were analysed by fluorescence spectroscopy with excitation/emission at 485 nm/535 nm. All experiments were conducted with 3 biological replicates.

### 2.10 Release of silver ions from PDA–Ag

The TMB assay is adapted from González-Fuenzalida *et al.*, 2013.^38^ 0.1 mg mL^−1^ PDA–Ag was exposed to microwaves for 0, 2, 5, 30 or 60 minutes. 20 μL of the sample was mixed with 0.1 M pH 4 NaAc/AcH buffer and TMB (10 mM in ethanol) in a 96-well plate and absorbance measured in the range 350–750 nm to highlight changes at 370 nm and 652 nm (charge transfer complex produced in the presence of Ag^+^).

### 2.11 Singlet oxygen detection

A 200 μL Eppendorf was filled with 40 μM *p*-nitrosodimethylaniline (RNO), 50 mM sodium phosphate buffer, 8 mM imidazole and 0.1 mg mL^−1^ PDA–Ag, and exposed to was exposed to microwaves for 0, 2, 5, 30 or 60 minutes. 100 μL was taken for UV-Vis spectroscopy at 440 nm in a 96-well plate.

### 2.12 Hydroxyl radical

A 200 μL Eppendorf was filled with 40 μM RNO, 50 mM sodium phosphate buffer and 0.1 mg mL^−1^ PDA–Ag, and exposed to was exposed to microwaves for 0, 2, 5, 30 or 60 minutes. 100 μL was taken for UV-Vis spectroscopy at 440 nm in a 96-well plate.

### 2.13 Hydrogen peroxide

Wells of a 96-well plate were filled with 100 μM Amplex Red, 0.25 μM horseradish peroxidase (HRP) and 0.01 M phosphate buffer (pH 7.4) to 100 μL. 0.1 mg mL^−1^ PDA–Ag with phosphate buffer (pH 7.4) was exposed to microwaves for 0, 2, 5, 30 or 60 minutes. 100 μL was added to the pre-prepared plates for fluorescence spectroscopy with excitation/emission of 571 nm/585 nm.

### 2.14 Statistical analysis

An asterisk indicates statistical significance (*p* < 0.05). Significant differences within groups were determined using one-way analysis of variance (ANOVA) followed by a multiple comparison analysis using Tukey and Bonferroni tests.

## 3. Results and discussion

### 3.1 Synthesis and characterisation of PDA–Ag NP

**Fig. 1:**
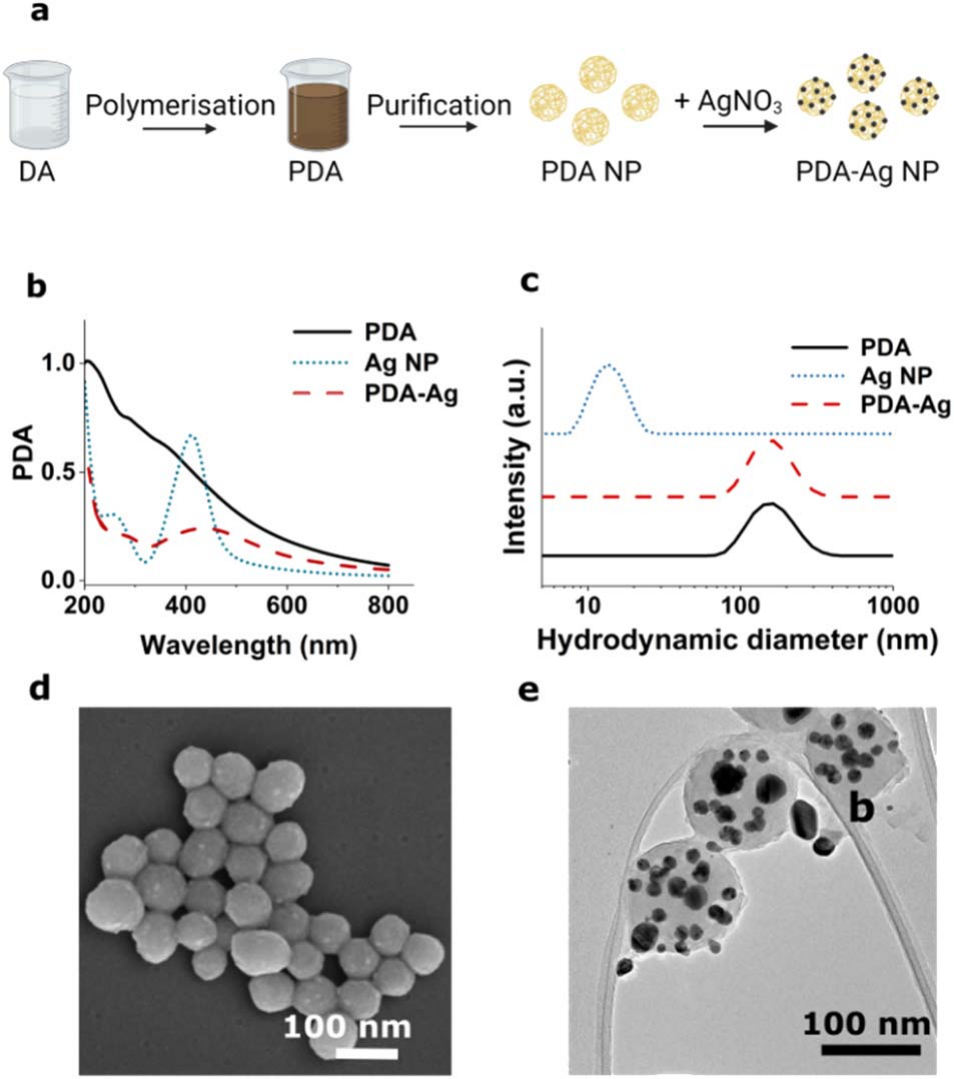
Synthesis and characterisation of PDA–Ag. (a) An overview of the synthesis of PDA–Ag, (b) UV-Vis spectroscopy of PDA, PDA–Ag and Ag NP, (c) hydrodynamic diameter information obtained by DLS of PDA, PDA–Ag and Ag NP, (d) SEM and (e) TEM images of PDA–Ag.

The PDA nanocarrier was synthesised according to established procedures employing aqueous ammonia, deionised water, ethanol, and dopamine hydrochloride to obtain PDA at a final concentration of 1 mg mL^−1^ (Experimental).^39,40^ Silver nitrate (AgNO_3_) was then added to the dispersion and reduced by phenolic units within PDA resulting in formation of Ag NPs on the surface of PDA spheres (Fig. 1), as previously described by Luo *et al.*^37,40^

The preparation of PDA–Ag was confirmed by UV-Vis spectroscopy (Fig. 1b), DLS (Fig. 1c), and scanning and transmission electron microscopy (SEM, TEM, Fig. 1d and e). The Ag NPs were found to have a broad absorption peak around 440 nm and TEM analyses an average size of 20 ± 4 nm. The spherical PDA carriers were found to have a size of 121 ± 11 nm prior, and 132 ± 19 nm after the growth of Ag NP. A zeta-potential of −21.3 ± 0.9 mV was obtained, which was lower than that of the 20 nm citrate-capped Ag NPs used as control (−38 ± 0.3 mV).

Prior to the assessment of their antibacterial activity, the impact of microwave exposure to PDA–Ag NPs was explored to estimate the level of any structural damage. To this end, UV-Vis, DLS, zeta-potential and TEM characterisation were performed after exposure to 1.3 W, 2.45 GHz microwaves in time intervals of 0, 2, 5, 30 and 60 minutes under pulsed excitation. The results obtained indicate there are no significant changes in size and the surface charge (as indicated by no differences in zeta potential) of the NPs upon microwave exposure (ESI Fig S1†).

### 3.2 Antibacterial activity of PDA–Ag in the absence of microwave irradiation

To explore the antibacterial activity of PDA–Ag and assess potential MW-induced enhancement, antibacterial activity studies using Gram-negative *Escherichia coli* (*E. coli*), and Gram-positive *Bacillus subtilis* (*B. subtilis*) bacteria were performed. In general, Gram-negative bacteria are more resistant to antimicrobial treatment due to their outer membrane structure.^38^

As an initial bactericidal study in the absence of microwaves, the zone of inhibition (modified Stoke's disc diffusion test) associated with exposure to PDA–Ag (0.2 mg mL^−1^) was assessed and compared to PDA (0.2 mg mL^−1^) and Ag NP (15 μg mL^−1^) controls. It should be noted that the amount of Ag NP control was matched to the amount of Ag determined in the PDA–Ag using inductively coupled plasma (ICP) analysis. Factors such as the size and surface charge of the nanoparticle, agar depth and composition, temperature and humidity all play a role in the resulting zone of inhibition. However, with the Stoke's disc diffusion method, all discs with the compounds of interest are placed on the same agar plate to eliminate environmental factors affecting the diffusion rate.^39^

**Fig. 2:**
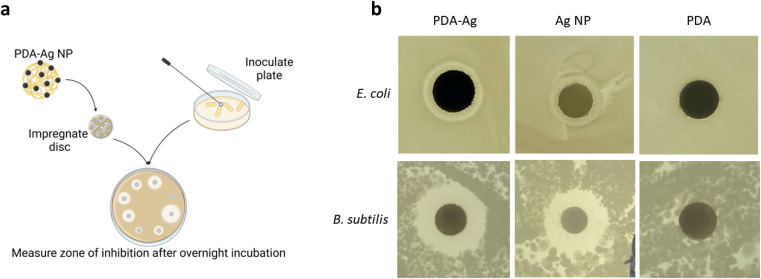
Zone of inhibition studies for PDA–Ag. (a) Overview of the experimental procedure for the zone of inhibition studies. (b) The zone of inhibition sizes for 0.2 mg mL^−1^ PDA–Ag, 15 μg mL^−1^ Ag NP and 0.2 mg mL^−1^ PDA on the same plate. This indicates 1 of 5 plates used to calculate the average inhibition zone. Discs are 6.5 mm in diameter.

As seen in Fig. 2b, both PDA–Ag (0.2 mg mL^−1^) and Ag NP (15 μg mL^−1^) are toxic to *E. coli* and *B. subtilis* as indicated by the inhibition zones larger than 3 mm. The inhibition zones of control, PDA NPs alone, were smaller than 2 mm, thus they can be considered non-toxic at studied concentration of 0.2 mg mL^−1^.

To determine the concentration at which PDA–Ag completely inhibits the growth of *E. coli* or *B. subtilis* over 24 hours in the absence of microwave irradiation, a minimum bactericidal concentration (MBC) assay was performed. Due to the scattering effects of PDA–Ag, the conventional assay exploiting the absorption characteristics of bacteria in solution to determine concentration (minimum inhibitory concentration) could not be performed. Instead, a plate count assay was conducted (ESI Fig S2†). The concentration of PDA–Ag which completely inhibits the growth of *E. coli* and *B. subtilis* in the absence of microwaves over a 24 h was found to be 50 μg mL^−1^ and 45 μg mL^−1^ respectively, consistent with previously reported values for Ag NPs.^40^ Note that MIC and MBC of Ag NPs can vary significantly (0.25 μg mL^−1^ to 621 μg mL^−1^) depending on size, shape and surface functionalisation.^41–43^

### 3.3 Microwave-enhanced antibacterial activity

The impact of microwave treatment was then explored first by using 1.3 W, 2.45 GHz microwaves and 0.1 mg L^−1^ of PDA–Ag. Microwaves of 2 W and below are considered low-power microwaves,^41–43^ and 2.45 GHz is the most used frequency band for industrial, scientific, and medical applications both for microwave heating (“thermal”) and excitation (“non-thermal”). A concentration of 0.1 mg mL^−1^ PDA–Ag was chosen as a suitable concentration for the study of MW impact, as a considerable level of toxicity was observed at 0.2 mg mL^−1^, even in the absence of microwaves. Thus, to assess the effect of MW radiation, lower concentration of PDA–Ag NPs should be employed.

Initially, the bactericidal activity was studied by incubating bacteria with 0.1 mg mL^−1^ PDA–Ag and taking aliquots at 0, 2, 5, 30 and 60 minutes in the absence and presence of microwave radiation. The aliquots were then plated onto LB agar and CFUs were counted after overnight incubation at 37 °C. In addition to CFU counting, ROS quantification was performed using cell penetrating probe, 2′,7′-dichlorofluorescein diacetate (DCFDA) to assess ROS produced within bacteria.

**Fig. 3:**
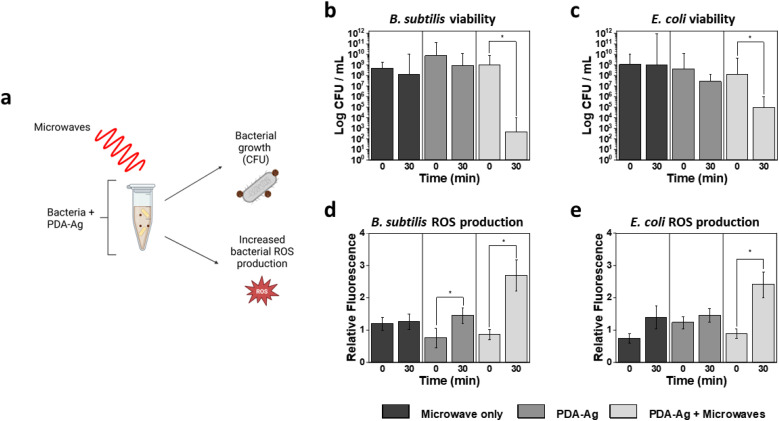
Bacterial cell viability and corresponding ROS production. Schematic diagram summarising (a) the experimental design, (b) cell viability of *B. subtilis*, (c) cell viability of *E. coli*, (d) bacterial ROS production for *B. subtilis*, (e) bacterial ROS production for *E. coli* before and after 30 min exposure to microwaves. The experiments were conducted with 3 biological replicates.

As shown in Fig. 3, bactericidal activity is significantly increased both in the case of *E. coli* and *B. subtilis* in the presence of PDA–Ag coupled with microwave treatment compared to PDA–Ag alone. A 3-log reduction in viability is observed when *E. coli* is exposed to both PDA–Ag and MW, indicating the activity-enhancing role of the MW exposure. Intracellular ROS analyses also showed significantly higher ROS production in those samples as compared to controls that included NP and MW treatment alone. Low-power MW were found to have negligible toxicity to bacteria, and no significant toxicity was observed for *E. coli* in the presence of PDA–Ag NPs within 30 minutes. However, although MW exposure does not have significant effect on the growth of *B. subtilis* colonies, 1-log reduction is observed in the presence of PDA–Ag NPs alone, and a large 4-log reduction when PDA–Ag are used in combination with MW.

Interestingly, when MW alone are applied to *E. coli* (ESI Fig S4†), an apparent increase in colony forming ability is observed within the first 5 min. The effect may be due to the disruption of cell clusters, causing an increase in CFU. However, some studies have also documented proliferative effects of sub-lethal amounts of ROS species for some bacteria.^44^ It is believed that small amounts of ROS can stimulated growth by activation of ROS-neutralising mechanisms such as overexpression of intrinsic antioxidants.^45^

However, despite this initial spike in colony forming ability after the first 5 min, extended microwave treatment results in destruction of bacterial cells, most likely due to the observed increase in intracellular ROS production and other mechanisms discussed below.

### 3.4 Possible mechanisms of MW-enhanced activity

Although detailed biomolecular profiling of observed effects was not in the scope of this study, we were interested in shedding some light onto the possible role of MWs in ROS production and explore any temperature effects that might contribute to the inactivation of bacteria.

Previously it was shown that MW-absorbing materials, including various nanomaterials, can be used in MW-guided thermal therapy in which local temperature increase is achieved through conversion of microwave energy into thermal energy.^41^ To explore microwave-induced heating of bacterial cultures, the temperature during the experiments was monitored using a thermocouple (ESI Fig S3a†). At higher concentrations of NPs (0.2 mg mL^−1^ PDA–Ag, 0.2 mg mL^−1^ PDA and 15 μg mL^−1^ Ag NP), we observed that samples containing PDA resulted in larger temperature increases (6.1 °C and 6.3 °C for PDA–Ag and PDA respectively) than samples containing Ag NP alone (1 °C) (ESI Fig S3b†). Although the temperature remains below those required for thermal inactivation of vegetative bacteria (>40 °C), we cannot exclude the possibility of a localised, larger temperature increase around the NPs in the proximity of the bacterial cells. Considering PDA is known for its adhesive properties,^46,47^ we also wanted to explore the possible attachment of PDA–Ag NPs to the bacterial cell envelope, which could result in formation of damaging hotspots.

**Fig. 4:**
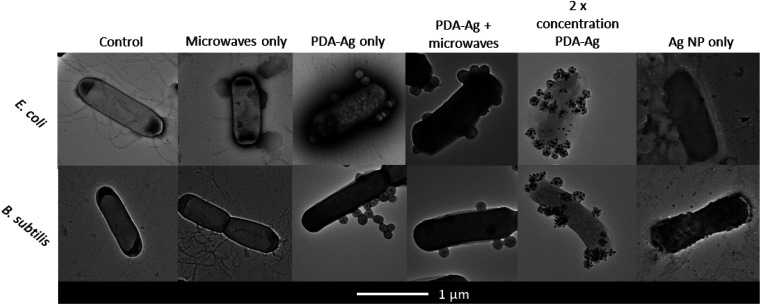
TEM images of PDA–Ag TEM images of *E. coli* and *B. subtilis* before and after exposure to microwaves in the presence of 0.1 mg mL^−1^ PDA–Ag, 0.2 mg mL^−1^ PDA–Ag and corresponding amount of Ag NP (7.5 μg mL^−1^).

As shown in Fig. 4, TEM images of bacteria treated with PDA–Ag and Ag NPs in presence and absence of microwaves were obtained. Microwave exposure alone did not result in any observable cell wall and membrane damage, which was expected as we are using low-power microwaves (1.3 W, 2.45 GHz), significantly below the power of 400–2000 W reported to cause pore formation and leakage.^25,48^ In the case of bacteria treated with PDA–Ag NPs, it could be observed that the NPs are aggregating at the surface of bacteria, but without causing any visible cell envelope damage. Bacteria treated with Ag NP in absence of MW exhibited signs of cellular leakage and some structural damage.

Considering the TEM images and obtained data, both the localised microwave-heating of PDA–Ag, non-thermal effects leading to membrane damage as well as possible accumulation of Ag^+^ ions within the bacterial envelope might lead to observed increased toxicity of PDA–Ag NPs under MW exposure. Previous studies in Gram-positive and Gram-negative bacteria have shown that the accumulation of Ag NP or Ag^+^ in the bacterial cell envelope is followed by the separation of the cytoplasmic membrane from the cell wall, which results in bacterial death.^49^

To explore whether there is any Ag^+^ released from the PDA–Ag system under microwave exposure, we used the 3,3′,5,5′-tetramethylbenzidine dye (TMB) assay, where a blue charge transfer complex (absorbance at 370 nm and 652 nm) is formed in presence of Ag^+^ ions.^38^

**Fig. 5:**
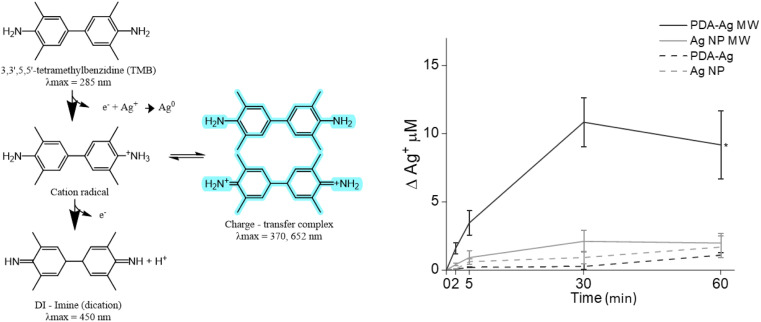
The detection of Ag^+^ released from PDA–Ag. TMB assay used for detection of released Ag^+^, showing Ag^+^ release over 60 minutes for PDA–Ag and Ag NP, with or without exposure to microwaves.

First, 0.1 mg mL^−1^ PDA–Ag was exposed to microwaves (1.3 W, 2.45 GHz) for 1 hour and the TMB essay performed by taking aliquots of the solution at regular intervals. As seen in Fig. 5, up to 10.8 μM (1.3 ppm) of Ag^+^ was released from PDA–Ag NP within 30 minutes of MW exposure, compared to a maximum of 2.1 μM released from Ag NP. According to previous reports, continuous exposure to 0.5 to 20 ppm of Ag^+^ ions is required to completely kill the bacteria overnight.^50–52^ Therefore, the measured concentration of Ag^+^ ions released from PDA–Ag NPs und MW exposure could not account for the high level of bacterial death.^53–56^ However, these ions might exert a localised effect if the release happens in a close proximity to the bacterial membrane. This might be the case with PDA–Ag NPs, which show adhesion to the bacterial surface.

**Fig. 6:**
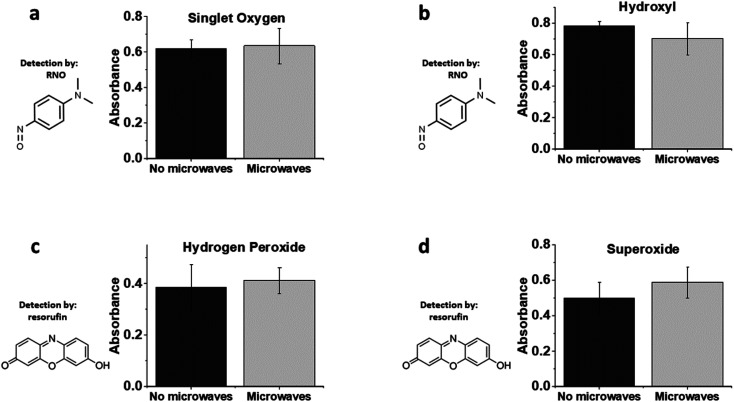
The production of reactive oxygen species from PDA–Ag. Relative absorbance of ROS indicators showing the amount of (a) singlet oxygen, (b) hydroxyl, (c) hydrogen peroxide and (d) superoxide produced in presence of 0.1 mg mL^−1^ PDA–Ag NPs in absence (black) and presence (grey) of microwaves. 1.3 W, 2.45 GHz microwaves were used over 1 h.

Finally, we were interested in the potential release of harmful ROS from PDA–Ag particles in the presence of MWs. Production of singlet oxygen was explored using the *p*-nitrosodimethylaniline (RNO)–imidazole singlet oxygen assay by monitoring RNO oxidation at 440 nm (ESI Fig S6†).^57,58^ In the absence of imidazole, the RNO assay can also be used for detection of hydroxyl radicals. As shown in Fig. 6, no significant amounts of singlet oxygen or hydroxyl radicals were produced by microwave irradiation of PDA–Ag at a power of 1.3 W, 2.45 GHz. This is not surprising, as any ROS produced would likely be scavenged by PDA, which is a known antioxidant. In addition, H_2_O_2_ measurements employing a resorufin-based assay indicated negligible production of H_2_O_2_ over 1 h of microwave exposure.

Taking these data into account, it can be concluded that extracellular ROS production from PDA–Ag exposed to low energy MWs are not involved in bacterial inactivation, although there is a significant amount of intracellular ROS produced indicating high levels of physiological stress. Further studies will investigate additional biomolecular signatures associated with bacterial envelope stress.

## 4. Conclusions

Non-thermal microwave-driven antibacterial activity of PDA–Ag nanoparticles using representative Gram negative (*E. coli*) and Gram positive (*B. subtilis*) bacteria was explored. A 3-log reduction of *E. coli*, and 5-log reduction of *B. subtilis* was obtained after 30 minutes exposure to low-power (1.3 W), low frequency (2.45 GHz) microwaves. PDA–Ag NPs were found to be more toxic in the presence of microwaves than Ag or PDA NPs controls and this toxicity correlates with an increased intracellular production of damaging ROS species. To avoid global thermal effects due to MW application, the temperature of solution was maintained below 37 °C during the study. In addition, no significant ROS production in dispersions of NPs using conventional ROS assays. Considering these data together with TEM analysis of bacteria in exposed to different NPs in the presence MWs, we hypothesise that membrane adhesion regulated by the PDA component of PDA–Ag NP composite leads to enhanced membrane damage through the non-thermal effects of MWs. The proximity of NPs might result in high localised concentrations of damaging Ag^+^ ions released from embedded Ag NPs, and possible high temperature hotspots around the MW-exposed PDA–Ag NP.

We demonstrate that PDA–Ag nanocomposites can be successfully used in conjunction with low-power MW for efficient inactivation of bacterial growth. Considering the ease of manufacture, low concentrations of NPs required and synergistic effects (adhesion, ion release, MW enhancement) of NP components, we believe that microwave-assisted antibacterial activity could have significant potential in the design of anti-fouling surface coatings and materials, as required for implantable devices and engineered tissue scaffolds. Further studies will focus on exploring the underlying biochemical and structural changes withing the bacterial cell to shed light onto the role of MW.

## Conflicts of interest

There are no conflicts to declare.

## Supplementary Material

RA-014-D3RA07543E-s001
