# Supplementary material for: Microwave-enhanced antibacterial activity of polydopamine–silver hybrid nanoparticles
Source: RSC Adv. 2024 Mar 11;14(12):8331–40. doi: 10.1039/d3ra07543e (PMC10926840; doi:10.1039/d3ra07543e)
Supplement: RA-014-D3RA07543E-s001 [file RA-014-D3RA07543E-s001.pdf]

Electronic Supplementary Material (ESI) for RSC Advances.
This journal is © The Royal Society of Chemistry 2024

Supplementary Information

Microwave-enhanced antibacterial activity of polydopamine-silver hybrid nanoparticles

Swetha Lingamgunta[*a], Yao Xiao[a], Heungjae Choi[b], Graham Christie[a], Ljiljana Fruk[&a]

[a] Department of Chemical Engineering and Biotechnology, University of Cambridge, Cambridge, UK

[b] School of Engineering, Cardiff University, CF24 3AA, UK

[&] Corresponding author

Email: lf389@cam.ac.uk

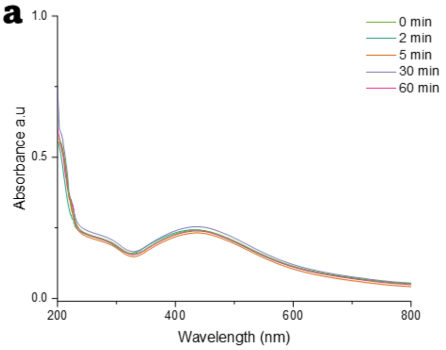

b

MW time (minutes)	DLS size (nm) ± PDI	Zeta Potential
0	216.7 ± 0.1	-21.3
2	209.3 ± 0.1	-18.0
5	201.3 ± 0.1	-20.0
30	182.6 ± 0.2	-23.7
60	197.9 ± 0.1	-19.2

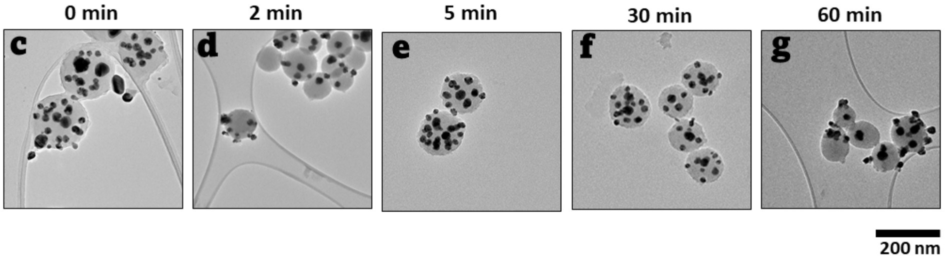

Fig S1. Impact of microwaves on PDA-Ag NPs. (a) UV-Vis spectra of PDA-Ag NPs after exposure to microwaves for 0 – 60 minutes. (**b)** Corresponding DLS size and zeta potential data of the samples are shown on the right. TEM images taken from sample at different intervals are shown in c-g.

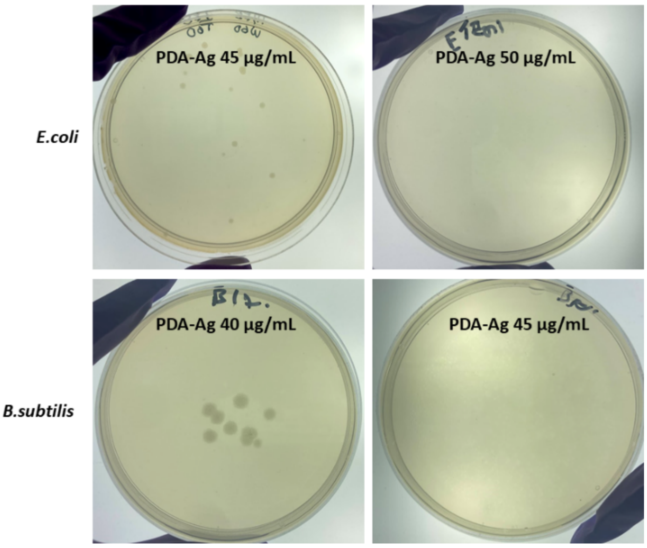

Fig S2. Minimum bactericidal concentration images. Pictures of agar plates (1 of 3 biological repeats) used to determine the MBC of PDA-Ag NPs for *E.coli* and *B.subtilis.*

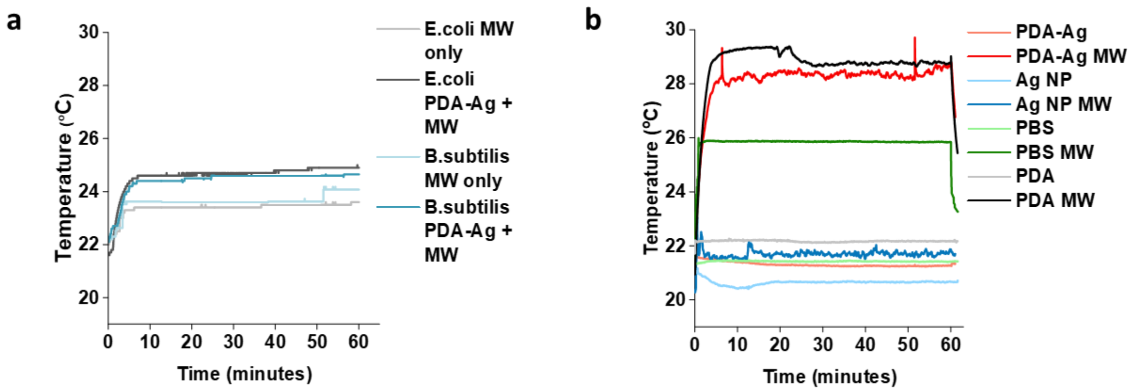

Fig S3 Temperature measurements of test solutions during experiments. (a) Temperature measurements during experiments (0.1 mg/mL PDA-Ag + 10^8 bacteria) to ensure temperature remains below thermal inactivation point. **(b)** Temperature measurements with 0.2 mg/mL PDA-Ag/PDA and PBS samples.

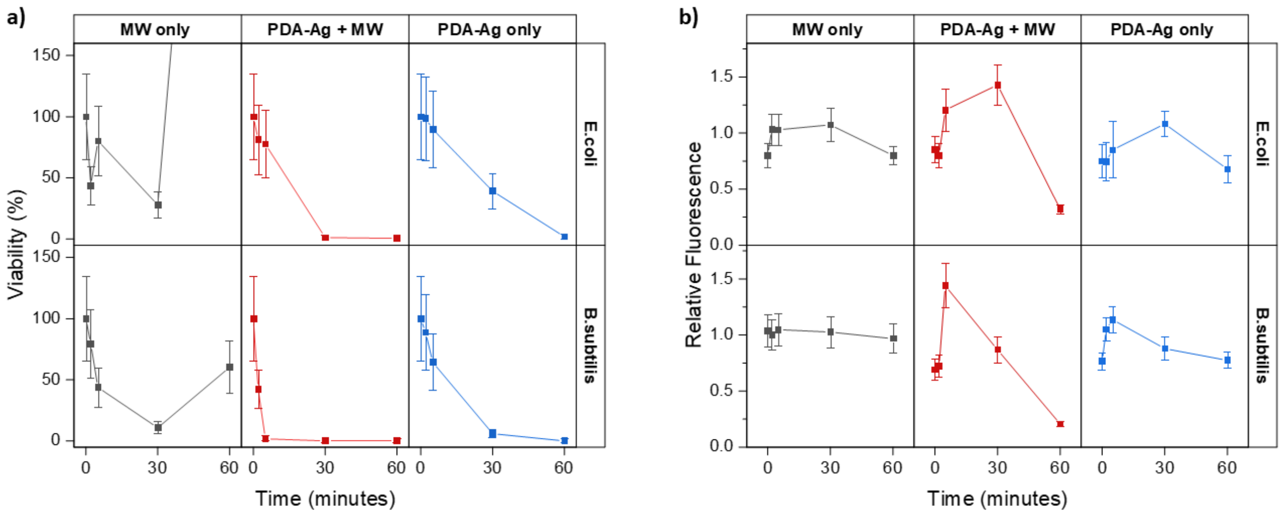

Fig S4. Bacterial cell viability and relative fluorescence of ROS indicators (showing bacterial ROS production). (a) Viability of *E.coli* and *B.subtilis* under microwave (MW) exposure, in presence of PDA-Ag NPs and MWs, and PDA-Ag NPs only. **(b)** Relative fluorescence of DCF (intracellular ROS indicator) for *E.coli* and *B.subtilis* in presence of microwaves (MW), PDA-Ag NPs and MW, and PDA-Ag NP only.

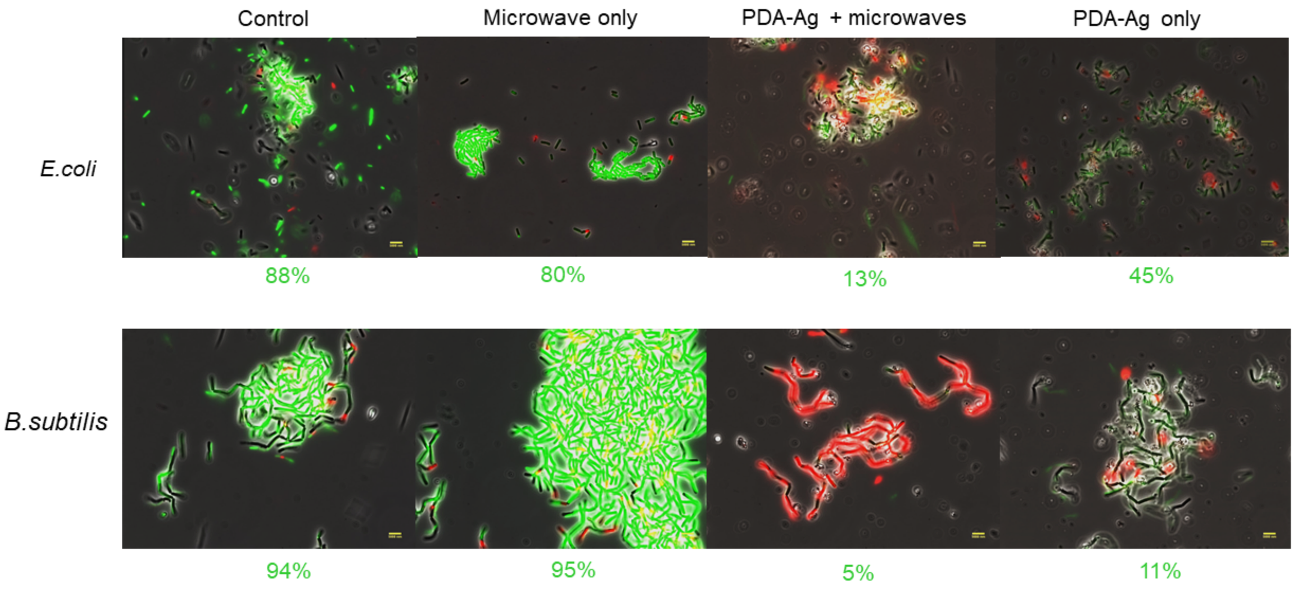

Fig S5. Live/dead assay images using SYTO 9 dye and propidium iodide staining. Fluorescent images showing *E.coli* and *B.subtilis*; control, microwave (MW) exposure, PDA-Ag and MW exposure or PDA-Ag only. SYTO 9 dye shows live cells in green, propidium iodide shows dead cells in red.

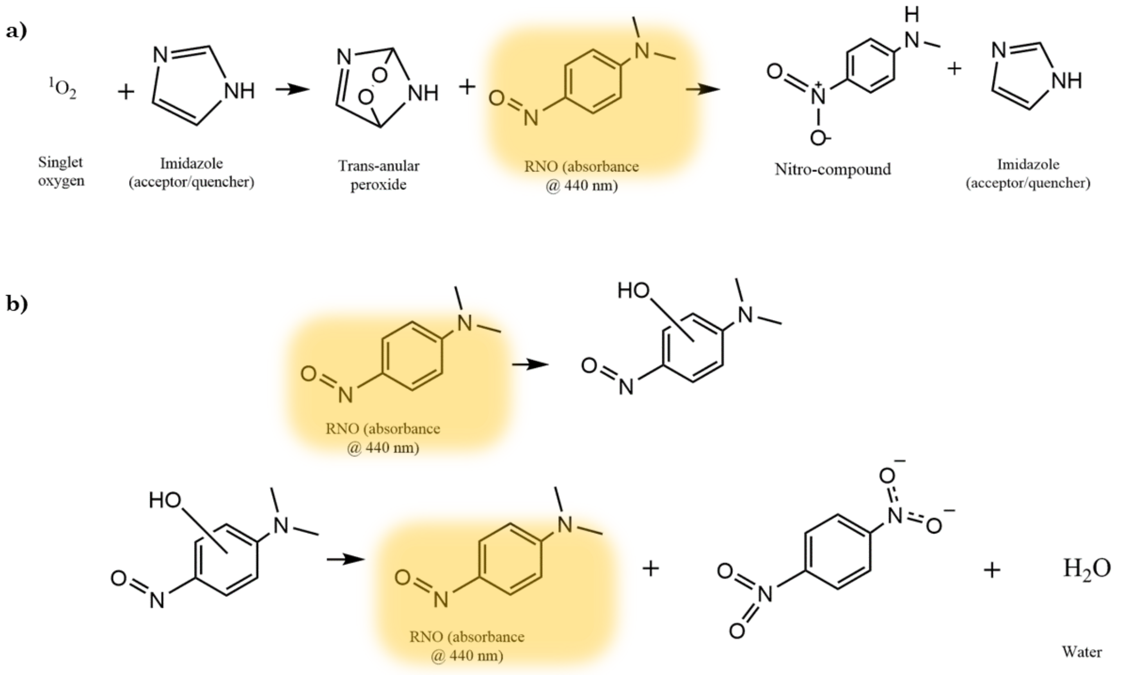

Fig S6. RNO assay used for the detection of singlet oxygen and hydroxyl. Mechanisms of **(a)** singlet oxygen and **(b)** hydroxyl detection using RNO.

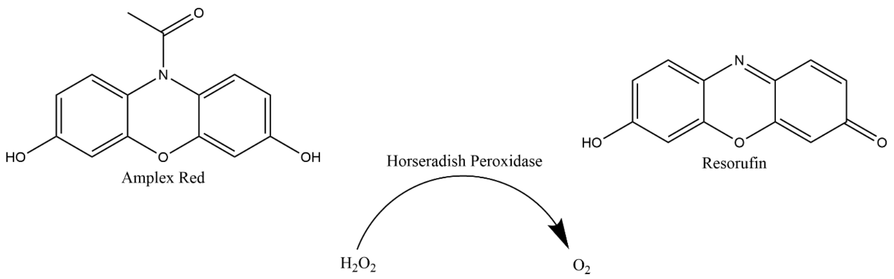

Fig S7. Amplex Red assay used for the detection of hydrogen peroxide and superoxide. Mechanism of hydrogen peroxide (H_2O_2) and superoxide sensing through the colorimetric detection of resorufin at 587 nm (with and without superoxide dismutase).
